
JOURNAL INFORMATION
==============================
NLM Title Abbreviation: Tex Med J (Austin)
NLM Journal ID: 21130590R

Texas Medical Journal

ISSN: 0892-8495
Publisher: Biomedical Journal Digitization Project

ARTICLE INFORMATION
==============================
PMCID: PMC9615744
Article version: 1
Subjects: Books and Magazines

A Treatise on Fractures and Dislocations

For Practitioners and Students

Print publication date: 1901 Sep
Volume: 17
Issue: 3
First page: 108
Last page: 108
License: This work is free of known copyright restrictions. For more information, please see PMC Back Issue Digitization.
License URL: https://creativecommons.org/publicdomain/mark/1.0/

==============================

MODERN MEDICINE. By Julius L. Salinger, M. D., Demonstrator of Clinical Medicine, Jefferson Medical College, etc., and Frederick J. Kalteyer, M. D., Assistant Demonstrator of Clinical Medicine, Jefferson Medical College, etc. Illustrated; pp. 801; price, cloth, $4.00; half morocco, $5.00. Philadelphia and London: W. B. Saunders & Co. 1900.

This book is not along beaten lines altogether. It presents the practice of internal medicine from the standpoint of a number of specialists, such as physical diagnosis, bacteriology, the examination of the gastric contents, the urine, the blood, the feces, etc. The practitioner of today must be familiar with all of the recent methods for arriving at conclusions in the practice. The man who does not spend some of his time in the laboratory is little less than a prescriber, a mere guesser. This work is particularly prominent in its teachings of the methods of the diagnosis and treatment. Hence the title—Modern Medicine. T. J. B.

---

A TREATISE ON FRACTURES AND DISLOCATIONS. For practitioners and students. By Lewis A. Stimson, B. A., M. D., Professor of Surgery in Cornell University Medical College, New York. New (3d) edition. In one octavo volume of 842 pages, with 336 engravings and 32 full-page plates. Cloth, $5.00 net. Leather, $6.00 net. Just ready. Lea Brothers & Co., Philadelphia and New York.

This book has shown much popularity. The second edition, which was a large one, was exhausted in one year. This, the third edition, goes forth with promise of still greater sales, on account of the thoroughness with which it has been revised, and increased size owing to additions in the text and illustrations.

This volume is divided into forty-eight chapters, and it is difficult to say which shows the greatest amount of care and research in its preparation. The introduction is devoted to definitions, statistics, influence of sex, age and season. Two chapters are devoted to pathology and etiology. Then follows a discussion of the early symptoms, diagnosis, repair, clinical causes, complications and remote consequences. The work throughout is practical and will meet with a large sale. T. J. B.

---

### Publisher's Department.

### Antikamnia and Heroin Tablets.

Our readers will find in this number the announcement of a new remedial preparation, viz.:—"Antikamnia and Heroin Tablets,"
